# The Great Drought of 1921 : Chlorination of Impure Water Supplies

**Published:** 1922-06

**Authors:** Alexander C. Houston


### THE GREAT DROUGHT OF 1921: CHLORINATION OF IMPURE WATER SUPPLIES.

By SIR ALEXANDER C. HOUSTON, K.B.E., C.V.O., M.B., D.Sc.

It used to be considered that the rainfall could never, even in the driest year, fall appreciably below 60 per cent. of the average. In the year 1921 it was almost exactly 60 per cent. in London, but there were large areas in the south-east of England where it was 50, and, indeed, in some cases only 40 per cent. of the average. The deficiency of rainfall was truly most remarkable, and this, combined with the brilliant sunshine and high temperature and evaporation, led to a serious shortage of water in a great many places.

The following table shows the temperature, rainfall and sunshine records for 1921 (Camden Square):—

Table Showing Temperature, Rainfall and Sunshine During Each Month of the Year 1921 (Camden Square).

| Month. | Mean temperature degrees F. | Rainfall in inches. | Duration of sunshine in hours. |
|---|---|---|---|
| Jan. ... | 46·0 | 2·48 | 20·0 |
| Feb. ... | 40·7 | 0·18 | 46·8 |
| March ... | 46·8 | 1·19 | 113·8 |
| April ... | 49·5 | 1·29 | 191·7 |
| May ... | 57·0 | 1·03 | 227·6 |
| June ... | 61·8 | 0·37 | 221·7 |
| July ... | 69·4 | 0·13 | 253·9 |
| Aug. ... | 63·7 | 1·65 | 157·3 |
| Sept. ... | 60·5 | 2·50 | 134·6 |
| Oct. ... | 56·2 | 0·63 | 130·4 |
| Nov. ... | 39·4 | 2·01 | 27·1 |
| Dec. ... | 44·4 | 1·13 | 25·0 |
| | | Total 14·59, a deficiency of 9·88 (60 per cent. of the average) | |

The position gradually became so acute that in some rural areas water had to be transported by rail or road for considerable distances, both for domestic purposes and to save the lives of animals. In many large towns the supply had to be seriously curtailed, and great inconvenience was caused in consequence. The damage from an agricultural and horticultural point of view was great. In several cases recourse had to be made to abandoned sources of supply, and new sources were tapped which, in the ordinary course of affairs, would not have been dreamt of. It is not a little remarkable that notwithstanding the grave depression that prevailed during 1921, the health of the people remained, so far as can be ascertained, wonderfully good. The only water-borne epidemic that seems to have occurred took place in May at Ogmore Vale, Mid-Glamorgan. Here, apparently as a result of tapping an auxiliary and impure source of supply, over 700 cases of dysentery occurred. Nevertheless, a period of great anxiety was passed through, and disastrous consequences might easily have resulted had the drought lasted only a little longer, either directly from acute shortage, or indirectly through the consequences of drinking impure water. In one Midland town of importance, to take a single example, the storage reservoirs were run bone dry, and the situation was only saved by boldly breaking into the gravel lower down the valley, and supplying chlorinated, but safe, water to the consumers. Some persons were possibly unduly pessimistic during 1921, but the childish optimism of others was lamentable. How near we were to a paralysis of trade and a stampede of terrified inhabitants from certain areas will never be known, but when a condition of practical drought covers eleven-twelfths of a year, and available sources of water supply are reaching the vanishing point, it requires no great effort of imagination to conjure up possibilities which are perhaps best not expressed in words.

It is an old saying that good may come out of evil, and certainly the drought of 1921 taught us many useful lessons. Not least was the evidence that we have been extremely wasteful in the past in the consumption of water, and that without infringing the unwritten laws of decency, or endangering health, the supply per head can be reduced to a very material extent. As regards 1922 the position is obscure, because we do not know how far we have borrowed on our natural reserves of underground water, nor can we be sure that the abnormal meteorological conditions which prevailed in 1921 are altogether things of the past. Otherwise the position is not unsatisfactory, for the rainfall in January and February was above, and in March nearly up to the average,* and full opportunity has been taken of every chance of impounding water in our previously depleted storage reservoirs. Nevertheless, we cannot expect January, February and March of 1922 to compensate for the failures of October, November and December, 1921, and we have now reached a period of the year when a variety of circumstances (luxuriant growth of vegetation, high temperature and evaporation) militate against the usefulness of wet weather for waterworks purposes. The pinch will probably come in the summer or autumn, and although it is hoped that all will be well even then, it is wise to prepare beforehand for all eventualities.

Beyond economy in the use of water, curtailment of the supply, and prevention of waste, there are no means of overcoming a shortage of water, except by utilising sources of supply which would not otherwise be considered safe for domestic use. Possibly one should add to this the remark that conditions may arise in the future causing some of our normal supplies to become so unsatisfactory in quality that remedial treatment may be strongly indicated. This leads me to a consideration of the main thesis of this article: *How may impure water be rendered safe for domestic consumption?*

As regards rural supplies, the following quotation (pages 35 and 36) is taken from "Rural Water

* At the time of writing this report (April 19), the rainfall has already appreciably exceeded the average for the whole month.

Supplies and their Purification" (John Bale, Sons & Danielsson, Ltd.):—

STERILISATION BY MEANS OF HEAT.

"This method does not aim directly at clarifying a water or eliminating taste, or purifying it chemically. Nevertheless, from the point of epidemic water-borne disease, it has no equal, and it differs from all other kinds of treatment, inasmuch as it is applicable to every kind of water (rain-water, river-water, surface-water, spring-water, well-water, &c.). The old rule was to boil a water violently for at least five minutes; this procedure was based partly on empiricism, and partly on a knowledge of the limitations of human nature. It has the disadvantage of robbing a water of most of its dissolved gases, and therefore rendering it less palatable. A perfectly safe rule to adopt is to bring three parts of water barely to boiling point (212°F., 100°C.), and add one part of "unboiled" water. The mixture within five minutes will be innocuous, whatever the temperature of the water was antecedently, and the dissolved gases will only be partly dissipated. For example, even with water initially at the freezing point, the temperature of the mixture would be 167°F. (75°C.) a temperature more than sufficient to kill the typhoid bacillus within five minutes."

Other methods of dealing with impure water in rural areas are dealt with in the same book at considerable length. Reference should also be made to Kershaw's book (pages 22 to 25) on "Sewage Purification and Disposal" (Cambridge University Press), in which, under the heading of "The Utilisation of Rain-water for Domestic Purposes," the author says:—

"It may not be out of place at this point to refer briefly to rain-water collected from the roofs of cottages and outbuildings as a drinking water supply for places where water is scarce. It seems strange that this source of water supply is not more frequently used in England. In Bermuda (Pop. in 1907, 21,000; rainfall about 48 in. p.a.) the only water supply is obtained from the rainfall falling on roofs and specially-constructed 'catches,' from which it flows into large tanks cut out of the limestone and rendered with Portland cement. There is no question that many rural districts might make far more use of rain-water for domestic purposes than is now the case."

Passing next to methods of purifying water supplied to large communities, there is no method in any emergency which can compete with chlorination.

THE CHLORINATION OF WATER.

The writer, in conjunction with Dr. McGowan, was the first (1905) to use chlorine for sterilisation purposes in connection with the Lincoln typhoid epidemic (over 1,000 cases and over 100 deaths). The results were very successful bacteriologically and epidemiologically, although complaints of taste were frequent and wide-spread. Of course, at that time practically nothing was known about the subject, and had the writer possessed then his present knowledge the taste troubles could doubtless have been averted. In 1908 Johnson started at Chicago the chlorination wave, which has since spread all over the United States, Canada and many other countries. Progress in this country has been slow, but this is not altogether a disadvantage, as the too rapid adoption of chlorination methods might yield unsatisfactory results and so bring the whole process into disrepute.

During the war chlorination was practised with great success (thanks to Horrocks and his colleagues), except as regards taste. It is probable that our Sanitary Officers were, rightly enough, more concerned with questions of absolute safety than with sentimental considerations. Nevertheless, the complaints were wide-spread and bitter. The writer has heard that our Tommies often asked: "Who was the bloke that invented this 'ere chlorination stunt?" The reply on one such an occasion is alleged to have been as follows: "Some say 'Orrocks and some say 'Ouston, but I think it must have been the —— Kaiser."

The subject may be considered under the following headings: (1) Bacteriological results; (2) Epidemiological considerations; (3) Questions of dose; (4) Questions of taste; (5) Methods of administration; and (6) Economical considerations.

(1) *Bacteriological Results.*—The spores of bacteria cannot be destroyed with practicable doses of chlorine. The object to be aimed at is to kill all the microbes of epidemic water-borne disease, and this is judged by the destruction of B. coli in the water. Post-chlorination multiplication of harmless water microbes, which almost invariably occurs, should create no feeling of alarm. On the contrary, it affords evidence that nothing noxious remains in the water, as otherwise multiplication could not possibly take place.

(2) *Epidemiological Considerations.*—Although it is generally conceded that the destruction of B. coli means a "safe" water epidemiologically, there are some persons who are sceptical, or who make certain reservations before accepting chlorination as an absolute security against water-borne disease. It would take too much space to enter into controversial questions here, but the writer dealt with the subject at great length in a paper entitled "B. Welchii, Gastro-Enteritis, and Water Supply" (*Engineering News Record*, Vol. 87, No. 12). The following is an extract, and if any reader wishes a reprint of the entire paper the writer will be pleased to send him a complimentary copy.

"The evidence that spores are, *through water supply*, instrumental in causing gastro-intestinal disorders rests, for the moment, on insecure foundations, and the writer cannot help feeling that in the presence of a chlorination process which really (and not merely nominally) destroys B. coli in water, the danger of disease occurring has been unduly exaggerated. In cases where B. coli is not killed, and therefore where other non-sporing bacteria of pathogenic sort escape destruction, the results may be different, but this, if it occurs, arises out of the imperfection of the chlorination process and is not, as the writer thinks, due to the presence of spores."

(3) *Questions of Dose.*—The ordinary range of dose, in terms of available chlorine, is from 1 in 1 million to 1 in 5 millions (0·2 in 1 million). It must be remembered that a gallon of water weighs 10 lb., and that chloride of lime contains only about 33⅓ per cent. of available chlorine. Thus a dose of 1 in 1 million means 10 lb. of liquid chlorine (costing about 3s. 4d.), and 30 lb. of chlorine of lime (costing about 4s. 4½d.) respectively per million gallons of water treated. In the case of well waters containing only traces of oxidisable matter, a dose of 1 in 4, or 1 in 5 millions, is usually sufficient, but with impure waters containing much oxidisable matter and many bacteria the dose may have to be increased to 1 in 1 million, or even more.

Although certain chemical tests (*e.g.*, amount of oxygen used up by permanganate within 5 minutes, or length of time a blue colour persists after the addition of the chlorine on testing with potassium iodide and starch solution) may be of considerable help in arriving at the correct dose, the final test must inevitably be the bacteriological one of absence of B. coli from the treated water. It goes without saying that, within certain limits, the longer the duration of contact the smaller the dose of chlorine necessary to sterilise the water, and the more likely are the results to be uniformly satisfactory.

(4) *Questions of Taste.*—This is an extremely difficult subject, and has been seriously neglected in the past. There is an old saying: "You may take a horse to the water, but you cannot force it to drink." It is indefensible, unless in an emergency, to ask people to drink water which has a disgusting taste. Once a prejudice has been created against chlorination by faulty dosing, &c., it is extremely difficult to gain afterwards the goodwill of the consumers. In this connection the writer may be pardoned for quoting from an article* by Professor Melville C. Whipple :—

"The London laboratories have done a great deal in times past to circumvent the persistence of after-tastes and odours in chlorinated waters, taking as a basic principle the belief that the sentiment of consumers on this point, while not based upon injury suffered from the drinking of such waters, is nevertheless something to be respected and not laughed out of court. The latter view has too often been the one adopted in this country, but it is bound to yield as greater recognition is forced of the importance of physical qualities. The practice of overdosing was probably never carried out to a more illogical end than it was in many cases with the American Expeditionary Forces in France. The notion that 'if a little is good, more is better,' too often dominated the practice of water disinfection, particularly in Lyster bags. The result was a water of repugnant taste and odour, which defeated the real objects in view, namely, proper water discipline on the part of the troops and the consumption of adequate quantities of water. A more moderate dose, such as was applied at water stations having chlorinating apparatus, would have given the protection and would not have sent back to America some two million men with preconceived ideas as to the unfitness of chlorinated water for drinking purposes."

There are two main tastes—the chlorinous, usually associated with an overdose, and the iodoform, which is often noticed when dealing with relatively pure waters and minor doses of chlorine. The chlorinous taste may be removed by reducing the dose, but there is always the possibility that in so doing the iodoform taste may be experienced. Alternatively, the dose may be increased and dechlorination (by means of sulphites or sulphurous acid gas, $SO_2$) practised. In this way the dose could actually be increased (although this would never be necessary) ten, a hundred, a thousand, or more times, yet after dechlorination the water would be quite tasteless. The iodoform taste can usually be remedied by increasing the dose, although here the danger presents itself of a chlorinous taste developing instead, and if this occurs resort must then be had to dechlorination. Alternatively, potassium permanganate (2-8 lb. per m.g.) may be used to remove the iodoform taste, and in most cases the results are wonderfully good. Where, however, a water contains very little oxidisable matter (*e.g.*, well water) it may fail, or succeed only at the expense of producing a water having an appreciable pink tinge. Once a water has developed the iodoform taste, dechlorination, in the writer's experience, is powerless to remove it.

(5) *Methods of Administration.*—Chlorine may be added to water, either as an alkaline hypochlorite solution (*e.g.*, chloros), or as liquid chlorine, or as a bleach solution prepared from chloride of lime. Space forbids any details being furnished here, but the subject is being dealt with in the writer's Sixteenth Annual Report (now in press).*

(6) *Economical Considerations.*—Chlorination is an extremely cheap way of purifying water. The present price of chloride of lime containing 33 per cent. of available chlorine is £16 6s. 8d. per ton (or 5¼d. per lb. of available chlorine). Liquid chlorine costs about 4d. per lb., and is, of course, of 100 per cent. strength.

In emergencies and during periods, like the present, of grave financial stringency, chlorination is a cheap method of purifying water of extreme value. In the piping times of peace, when the world is again prosperous, its usefulness in emergencies remains the same, but its value in other cases is perhaps less attractive, unless viewed as an adjunct to more natural processes of water purification.

* March issue of the "Journal of the American Waterworks Association."

* The Metropolitan Water Board's agents for the sale of their publications are Messrs. S. P. King & Son, 2 and 4, Gt. Smith Street, Westminster, S.W.

